# (*R*)-[^11^C]verapamil is selectively transported by murine and human P-glycoprotein at the blood–brain barrier, and not by MRP1 and BCRP

**DOI:** 10.1016/j.nucmedbio.2013.05.012

**Published:** 2013-10

**Authors:** Kerstin Römermann, Thomas Wanek, Marion Bankstahl, Jens P. Bankstahl, Maren Fedrowitz, Markus Müller, Wolfgang Löscher, Claudia Kuntner, Oliver Langer

**Affiliations:** a Department of Pharmacology, Toxicology and Pharmacy, University of Veterinary Medicine, and Center for Systems Neuroscience, Hannover, Germany; b Department of Clinical Pharmacology, Medical University of Vienna, Austria; c Health and Environment Department, Biomedical Systems, AIT Austrian Institute of Technology GmbH, Seibersdorf, Austria; d Department of Nuclear Medicine, Preclinical Molecular Imaging, Hannover Medical School, Hannover, Germany

**Keywords:** Positron emission tomography, (*R*)-[^11^C]verapamil, Blood–brain barrier, P-glycoprotein, Multidrug resistance protein 1, Breast cancer resistance protein

## Abstract

**Introduction:**

Positron emission tomography (PET) with [^11^C]verapamil, either in racemic form or in form of the (*R*)-enantiomer, has been used to measure the functional activity of the adenosine triphosphate-binding cassette (ABC) transporter P-glycoprotein (Pgp) at the blood–brain barrier (BBB). There is some evidence in literature that verapamil inhibits two other ABC transporters expressed at the BBB, i.e. multidrug resistance protein 1 (MRP1) and breast cancer resistance protein (BCRP). However, previous data were obtained with micromolar concentrations of verapamil and do not necessarily reflect the transporter selectivity of verapamil at nanomolar concentrations, which are relevant for PET experiments. The aim of this study was to assess the selectivity of verapamil, in nanomolar concentrations, for Pgp over MRP1 and BCRP.

**Methods:**

Concentration equilibrium transport assays were performed with [^3^H]verapamil (5 nM) in cell lines expressing murine or human Pgp, human MRP1, and murine Bcrp1 or human BCRP. Paired PET scans were performed with (*R*)-[^11^C]verapamil in female FVB/N (wild-type), *Mrp1^(−/−)^*, *Mdr1a/b^(−/−)^*, *Bcrp1^(−/−)^* and *Mdr1a/b^(−/−)^Bcrp1^(−/−)^* mice, before and after Pgp inhibition with 15 mg/kg tariquidar.

**Results:**

In vitro transport experiments exclusively showed directed transport of [^3^H]verapamil in Mdr1a- and MDR1-overexpressing cells which could be inhibited by tariquidar (0.5 μM). In PET scans acquired before tariquidar administration, brain-to-blood ratio (*K*_b,brain_) of (*R*)-[^11^C]verapamil was low in wild-type (1.3 ± 0.1), *Mrp1^(−/−)^* (1.4 ± 0.1) and *Bcrp1^(−/−)^* mice (1.8 ± 0.1) and high in *Mdr1a/b^(−/−)^* (6.9 ± 0.8) and *Mdr1a/b^(−/−)^Bcrp1^(−/−)^* mice (7.9 ± 0.5). In PET scans after tariquidar administration, *K*_b,brain_ was significantly increased in Pgp-expressing mice (wild-type: 5.0 ± 0.3-fold, *Mrp1^(−/−)^*: 3.2 ± 0.6-fold, *Bcrp1^(−/−)^*: 4.3 ± 0.1-fold) but not in Pgp knockout mice (*Mdr1a/b^(−/−)^* and *Mdr1a/b^(−/−)^Bcrp1^(−/−)^*).

**Conclusion:**

Our combined in vitro and in vivo data demonstrate that verapamil, in nanomolar concentrations, is selectively transported by Pgp and not by MRP1 and BCRP at the BBB, which supports the use of (*R*)-[^11^C]verapamil or racemic [^11^C]verapamil as PET tracers of cerebral Pgp function.

## 1 Introduction

Positron emission tomography (PET) with [^11^C]verapamil, either in racemic form or in form of the (*R*)-enantiomer ((*R*)-[^11^C]verapamil), has been frequently used to non-invasively study the functional activity of the adenosine triphosphate-binding cassette (ABC) transporter P-glycoprotein (Pgp) at the blood–brain barrier (BBB) of animals and humans (see, for instance, Refs. [1], [2], [3], [4], [5], [6], [7]). In some studies, Pgp function was modulated by administration of Pgp inhibitors, such as cyclosporine A, valspodar or tariquidar [3], [4], [5], [6]. Moreover, PET with [^11^C]verapamil has been used to assess changes in Pgp function at the BBB occurring in neurological disorders, such as therapy-refractory epilepsy [8], Alzheimer's disease [9] or Parkinson's disease [10]. Surprisingly, despite its frequent use as PET tracer of cerebral Pgp function, the selectivity of [^11^C]verapamil among different ABC transporters expressed at the BBB has not been assessed before. Knowledge about the ABC transporter selectivity of [^11^C]verapamil is important, as there is a risk that PET results might be misinterpreted due to contribution of other ABC transporters than Pgp to [^11^C]verapamil efflux at the BBB.

Verapamil is at micromolar concentrations an inhibitor of Pgp [11]. At nanomolar concentrations verapamil is transported by Pgp [12], [13], [14], [15] thereby enabling PET measurement of Pgp function with tracer doses of [^11^C]verapamil. It has been suggested that verapamil is not a competitive inhibitor of Pgp but that Pgp inhibition is mediated via another binding site than transport leading to concentration-dependent transport of verapamil [15].

There is some evidence in the literature that verapamil also inhibits at micromolar concentrations other ABC transporters than Pgp, i.e. multidrug resistance protein 1 (humans: MRP1, rodents: Mrp1) [16], [17], [18] and breast cancer resistance protein (humans: BCRP, rodents: Bcrp1) [19]. Although verapamil itself does not appear to be transported by MRP1 [17], one study found that the fluorescent probe bodipy-FL-verapamil is a MRP1 substrate [20].

In many cases in vitro transport data are obtained using micromolar (i.e. therapeutically relevant) concentrations of a transporter substrate and do not necessarily reflect the transporter selectivity profile of a compound at nanomolar concentrations, which are relevant for in vivo PET experiments. We and others have for instance shown that in nanomolar concentrations the Pgp inhibitors tariquidar and elacridar are transported by Pgp and Bcrp1 at the murine BBB, but that transport is significantly reduced when the compounds are given in higher doses, most likely because they dose-dependently inhibit their own transport at the BBB [21], [22]. Tariquidar was found to be a dual Pgp/Bcrp1 substrate in nanomolar and a Bcrp1-selective substrate in micromolar concentrations [21], [23].

The aim of this study was to assess the selectivity of verapamil for Pgp over MRP1 and BCRP, in nanomolar concentrations, which are relevant for PET experiments. We used a combination of in vitro transport assays using cell lines stably expressing murine or human ABC transporters and in vivo PET experiments with (*R*)-[^11^C]verapamil in wild-type and transporter knockout mice before and after Pgp inhibition with tariquidar. By using this experimental approach we were able to demonstrate that (*R*)-[^11^C]verapamil is selectively transported by Pgp, and not by MRP1 and BCRP at the BBB.

## 2 Materials and methods

### 2.1 Chemicals

If not stated otherwise substances were purchased from Sigma-Aldrich Chemie GmbH (Schnelldorf, Germany). [^3^H]verapamil and [^14^C]mannitol (specific activity: 2.22–3.14 GBq/μmol and 1.85–2.22 kBq/μmol, respectively) were obtained from Hartmann Analytic GmbH (Braunschweig, Germany) and [^14^C]2-amino-1-methyl-6-phenylimidazo[4,5-b]pyridine ([^14^C]PhIP) (specific activity: 1.85 MBq/μmol) and PhIP from Toronto Research Chemicals (Toronto, Canada). Tariquidar dimesylate was obtained from Xenova Ltd. (Slough, UK) and MK571 from Alexis Biochemicals (Axxora, Lörrach, Germany). For in vivo experiments, tariquidar was freshly dissolved prior to each administration in 2.5% (wt/vol) aqueous dextrose solution and injected intravenously (i.v.) at a volume of 4 mL/kg. For the in vitro experiments, tariquidar and Ko143 were dissolved in DMSO (< 0.1% DMSO in final solution) and MK571 was dissolved in serum-free Opti-MEM® (Gibco®/Life Technologies Corporation, Darmstadt, Germany).

### 2.2 Cell lines

For the in vitro transport studies LLC-PK1 cells transduced with human *MDR1* or murine *Mdr1a* and respective wild-type LLC cells were used. Additionally, Madin-Darby canine kidney type II (MDCK-II) cells transduced with murine *Bcrp1* or human *BCRP* or human *MRP1* and respective wild-type MDCK-II cells were used. The cell lines were a kind gift from Piet Borst and Alfred Schinkel (The Netherlands Cancer Institute, Amsterdam, the Netherlands). Culturing of the cells was performed as described earlier [24]. MDR1- and Mdr1a-overexpressing LLC cells were regularly tested for vincristine sulfate resistance (0.64 μM) as described before [24]. The MDCK-II cells transduced with *Bcrp1* or *BCRP* were regularly tested for mitoxantrone resistance (20 μM).

### 2.3 Animals

Female FVB/N (wild-type), *Mrp1^(−/−)^*, *Mdr1a/b^(−/−)^*, *Bcrp1^(−/−)^* and *Mdr1a/b^(−/−)^Bcrp1^(−/−)^* mice were obtained from Taconic Inc. (Germantown, NY, USA). Mice were housed in groups of up to five individuals under controlled environmental conditions (22 ± 1 °C, 40%–70% humidity) with a 12-h light–dark cycle (lights on at 6:00) and ad libitum access to food and water. Mice underwent PET scans at a weight of 28.2 ± 0.6 g. The study was approved by the local animal welfare committee (Amt der Niederösterreichischen Landesregierung) and all study procedures were performed in accordance with the European Communities Council Directive of November 24, 1986 (86/609/EEC). Every effort was made to minimize both the suffering and the number of animals used in this study.

### 2.4 Concentration equilibrium transport assay (CETA)

CETA was performed in triplicate as described previously [25]. With a density of 0.3 × 10^6^ cells/cm^2^ LLC cells were seeded on transparent polyester membrane filters (Transwell-Clear®, 6-well, 24-mm diameter, 0.4-μm pore size, Corning Costar Corporation, Cambridge, MA, USA). With a density of 0.4 × 10^6^ cells/cm^2^ MDCK-II cells were seeded on translucent polyester membrane filters (ThinCert^TM^, 6-well, 24.85-mm diameter, 0.4-μm pore size, Greiner Bio-One, Frickenhausen, Germany). Transport experiments were performed 5–7 days after the cells reached 100% confluence. For 1 h pre-incubation, culture medium was replaced by serum-free Opti-MEM® with or without inhibitor (0.1 μM Ko143 for Bcrp1/BCRP inhibition, 50 μM MK571 for MRP1 inhibition or 0.5 μM tariquidar for Pgp inhibition). For experiments with MDCK-II cells tariquidar (0.2 μM) was added for inhibition of endogenous Pgp. Incubation was performed with [^3^H]verapamil in fresh Opti-MEM® on the apical and basolateral sides of the monolayer at a concentration of 5 nM. Samples were taken from both compartments after 60, 120, 240 and 360 min and the amount of [^3^H]verapamil was quantified with a β-scintillation-counter. Control CETA was performed with calcein AM (1 μM) for MRP1 [26] and [^14^C]PhIP (2 μM) for Bcrp1/BCRP. The membrane integrity was determined by mannitol diffusion and transepithelial electrical resistance (TEER), with criteria for exclusion described earlier [25].

### 2.5 Radiotracer synthesis and formulation

(*R*)-[^11^C]verapamil was synthesized as described previously [27] and formulated for i.v. injection in phosphate-buffered saline (pH = 7.4)/ethanol (9/1, vol/vol). Radiochemical purity, as determined by radio high-performance liquid chromatography, was greater than 98%, and specific activity at the end of synthesis was > 100 GBq/μmol.

### 2.6 Small-animal PET imaging and PET data analysis

Isoflurane anesthesia (1%–2% in oxygen) was induced and maintained during the whole experimental procedure. Mice were placed on a dual-animal bed which was kept at 38 °C to prevent hypothermia. A catheter, placed into a lateral tail vein, was used for i.v. administration of (*R*)-[^11^C]verapamil and tariquidar. At the beginning of (*R*)-[^11^C]verapamil administration, dynamic PET imaging was initiated using a microPET Focus220 scanner (Siemens Medical Solutions, Knoxville, TN, USA). Before the first PET scan, a transmission scan using a ^57^Co point source was recorded over 10 min.

Groups of FVB/N (wild-type), *Mrp1^(−/−)^*, *Bcrp1^(−/−)^*, *Mdr1a/b^(−/−)^* and *Mdr1a/b^(−/−)^Bcrp1^(−/−)^* mice (*n* = 3–5 per group) underwent paired PET scans with (*R*)-[^11^C]verapamil (injected activity: 1360 ± 74 MBq/kg in a volume of approximately 0.1 mL, corresponding to < 14 nmol/kg unlabeled (*R*)-verapamil) before and after administration of tariquidar (15 mg/kg; equivalent to 18 μmol/kg) (see Fig. 2 for diagram of study timeline). For this dose of tariquidar we have shown complete inhibition of cerebral Pgp in an earlier study in rats [7]. One hour after starting scan 1, tariquidar was i.v. administered over a time period of about 1 min, followed by 90 min of PET data acquisition. Scan 2 was started at 2 h after administration of tariquidar.

As blood sampling was not feasible during the paired PET scans, separate groups of each mouse type (*n* = 4–6 per group) were injected under isoflurane-anesthesia with (*R*)-[^11^C]verapamil (1210 ± 11 MBq/kg) before and after administration of tariquidar (15 mg/kg) and venous blood was sampled at 25 min after each (*R*)-[^11^C]verapamil injection by retro-orbital puncture. Blood samples were weighed and measured for radioactivity in a gamma counter. Blood radioactivity data were corrected for radioactive decay and expressed as standardized uptake value (SUV = (radioactivity per mL/injected radioactivity) × body weight). PET images were reconstructed by Fourier re-binning followed by two-dimensional filtered back projection with a ramp filter. A standard data correction protocol (normalization, attenuation and decay correction) was applied. Whole brain was manually outlined on PET images and time–activity curves expressed in SUV units were derived. Brain-to-blood ratios of activity (*K*_b,brain_) were calculated by dividing brain activity concentrations measured with PET at 25 min after (*R*)-[^11^C]verapamil injection by mean blood activity concentrations measured with a gamma counter in separate groups of mice.

### 2.7 Statistical analysis

Statistical analyses were performed using Prism5 software (GraphPad Inc, La Jolla, CA, USA). Data were statistically analyzed by two-way analysis of variance (ANOVA) for CETA and by one-way ANOVA for PET, followed by Bonferroni test to compare replicate means (CETA) or for selected pairs of columns (PET). Tests were used two-tailed and a *p* < 0.05 was considered statistically significant. If not stated otherwise, all values are given as mean ± standard error of the mean (SEM).

## 3 Results and discussion

In order to assess the ABC transporter selectivity of verapamil we performed transport experiments with [^3^H]verapamil in cell lines expressing murine or human Pgp, human MRP1, and murine Bcrp1 or human BCRP, with and without transporter inhibition by different inhibitors, as well as PET experiments with (*R*)-[^11^C]verapamil in wild-type and transporter knockout mice. A similar approach has been previously used to assess the ABC transporter selectivity of other ABC transporter PET tracers, i.e. [^11^C]tariquidar, [^11^C]elacridar [21] and [^11^C]-*N*-desmethyl-loperamide [28].

### 3.1 In vitro transport experiments

**Fig. 1:**
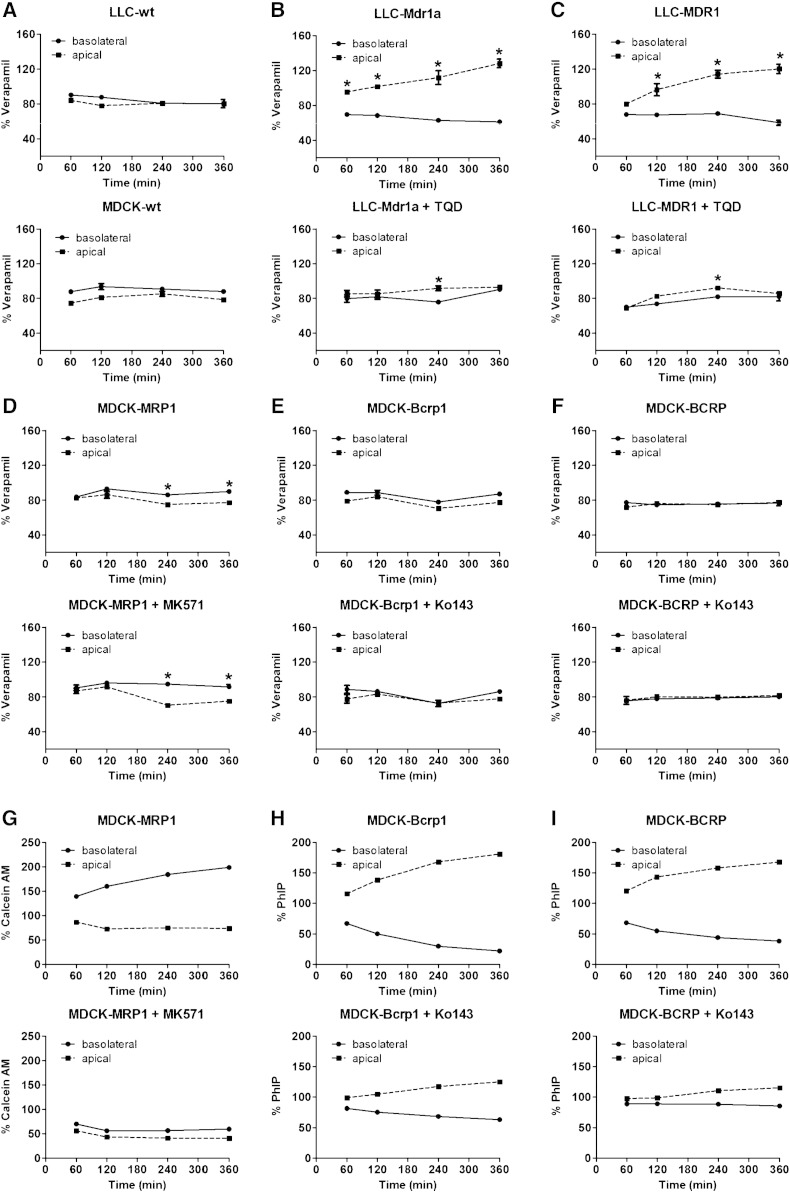
CETA with [^3^H]verapamil (5 nM) in wild-type (wt) LLC and MDCK cells (A), LLC cells transduced with murine *Mdr1a* (B) or human *MDR1* (C) and MDCK cells transduced with human *MRP1* (D), murine *Bcrp1* (E) or human *BCRP* (F) without and with respective inhibitors (tariquidar for Pgp, MK571 for MRP1 and Ko143 for Bcrp1/BCRP). In all experiments in MDCK cells tariquidar (0.2 μM) was added to inhibit endogenous Pgp. Additionally, control CETA was performed with calcein AM (1 μM) as a substrate for MRP1 (G) and [^14^C]PhIP (2 μM) as a substrate for Bcrp1 (H) and BCRP (I). Except for control experiments (*n* = 1), data are shown as the mean (± SEM, *n* = 3) of % initial verapamil concentration in the apical and basolateral chamber versus time. Significant differences are indicated by asterisk (*p < 0.05).

As it has previously been demonstrated that the conventional bidirectional transport assay may often fail to identify highly permeable compounds as transporter substrates due to their passive back-diffusion into the donor compartment, we employed CETA, in which the influence of passive diffusion is minimized by adding the drug at equal concentrations to the apical and the basolateral compartments [25]. As there is evidence that some transporter substrates may show different transporter selectivity profiles at nanomolar as compared with micromolar concentrations—most likely due to concentration-dependent transporter inhibition [14], [15], [21], [22], [29]—we used nanomolar (5 nM) concentrations of [^3^H]verapamil in CETA which are comparable to the concentrations achieved in vivo in PET experiments in blood and in tissue. As (*R*)-[^3^H]verapamil was not commercially available we used racemic [^3^H]verapamil in CETA. Wild-type LLC and MDCK cells showed no directional transport of [^3^H]verapamil (Fig. 1A). In contrast, Mdr1a- and MDR1-overexpressing cells showed significant transport of [^3^H]verapamil, which could be almost completely inhibited with tariquidar (0.5 μM) (Fig. 1B, C). In MRP1-overexpressing cells the concentration of [^3^H]verapamil was significantly decreased in the apical compared to the basolateral compartment, but this was not influenced by the MRP inhibitor MK571, indicating that this effect was not due to MRP1-mediated transport (Fig. 1D). Control CETA performed with calcein AM indicated that functional MRP1 was present and sensitive to inhibition with MK571 (Fig. 1G). It should be noted that one previous study found that the (*R*)- and (*S*)-enantiomers of verapamil had different effects on MRP1 activity [18]. Whereas (*R*)-verapamil was shown to directly inhibit MRP1, the (*S*)-enantiomer modulated MRP1 activity indirectly by stimulating MRP1-mediated glutathione transport [18]. In the present study, the use of racemic [^3^H]verapamil precluded the detection of possible differences in MRP1 interaction between (*R*)- and (*S*)-verapamil. No significant transport of [^3^H]verapamil could be detected in Bcrp1- and BCRP-overexpressing cells (Fig. 1E, F). Control experiments with [^14^C]PhIP demonstrated that Bcrp1 and BCRP were functional (Fig. 1H, I).

### 3.2 In vivo PET experiments

**Fig. 2:**
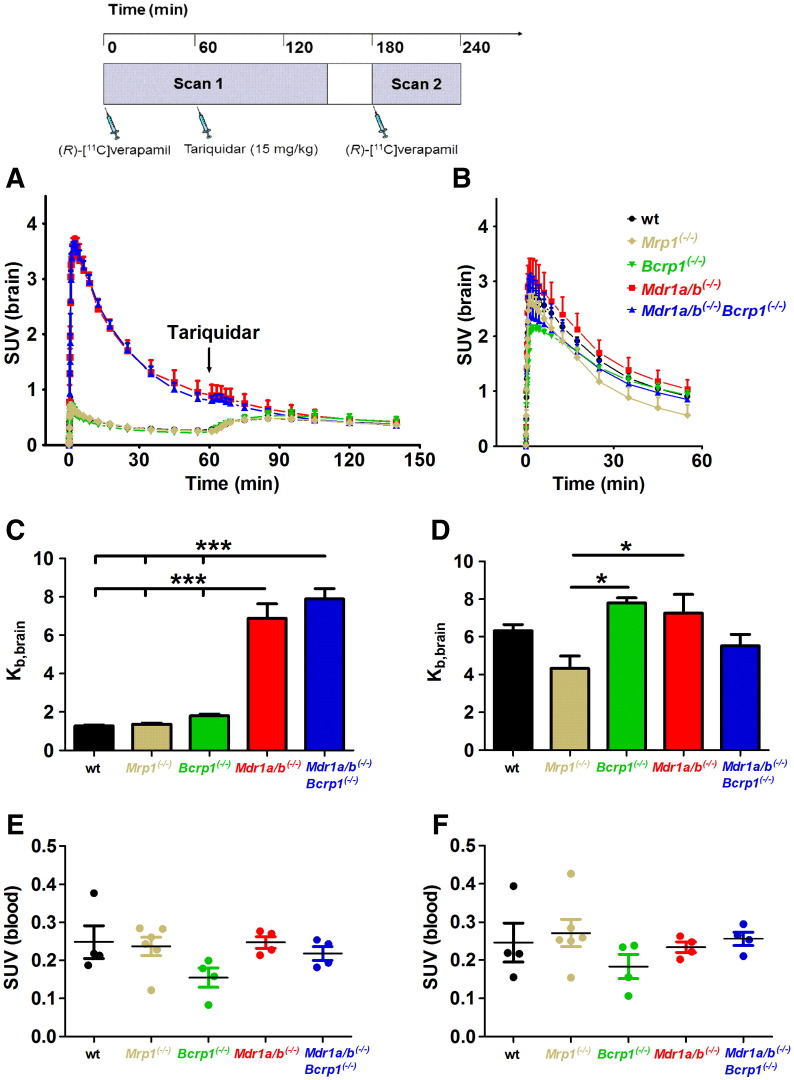
Mean (± SEM) whole-brain time–activity curves (SUV) of (*R*)-[^11^C]verapamil in wild-type (wt, *n* = 5, black circles), *Mrp1^(−/−)^* (*n* = 4, beige diamonds), *Bcrp1^(−/−)^* (*n* = 3, green triangles), *Mdr1a/b^(−/−)^* (*n* = 3, red squares) and *Mdr1a/b^(−/−)^Bcrp1^(−/−)^* mice (*n* = 3, blue triangles) for scan 1 (A) and scan 2 (B). At 60 min after start of scan 1 tariquidar (15 mg/kg) was administered as an i.v. bolus, which is indicated by an arrow. Scan 2 was performed at 2 h after administration of tariquidar (see timeline on top of the figure). In panels C and D, mean (± SEM) brain-to-blood ratios of activity (*K*_b,brain_) at 25 min after (*R*)-[^11^C]verapamil injection are shown for different mouse types before (C) and after (D) tariquidar administration. Blood activity concentrations (SUV) measured at 25 min after (*R*)-[^11^C]verapamil injection in separate groups of mice before and after tariquidar administration are shown in panels E and F, respectively. Significant differences are indicated by asterisk (*p < 0.05; ***p < 0.001).

As a second approach to investigate transporter selectivity of verapamil we performed PET experiments with (*R*)-[^11^C]verapamil in wild-type and transporter knockout mice before and after administration of tariquidar at a dose which completely inhibits Pgp at the BBB (Fig. 2) [7]. We have shown before that tariquidar does not influence (*R*)-[^11^C]verapamil metabolism and plasma protein binding [6]. We used a previously described paired scan protocol [6], in which tariquidar was administered *during* the first PET scan (i.e. at 60 min after injection of (*R*)-[^11^C]verapamil, see Fig. 2), which allows for directly studying the effect of tariquidar on (*R*)-[^11^C]verapamil brain kinetics [5]. To the best of our knowledge brain distribution of [^11^C]verapamil has not been assessed before in other ABC transporter knockout mice than Pgp knockout mice [1], [2].

Even though recent quantitative proteomics data indicate that MRP1 expression levels are below the limit of quantification at the murine and human BBB [30], some studies have obtained evidence for presence of functional Mrp1 activity at the murine BBB [31], [32]. MRP1 is also expressed at the blood–cerebrospinal fluid barrier, where it transports its substrates from cerebrospinal fluid into blood [33]. Human PET studies with (*R*)-[^11^C]verapamil have shown high uptake of radioactivity in the choroid plexus and the ventricular system [8]. However, due to the limited spatial resolution of small-animal PET (~ 1.5 mm) we were in the present study not able to analyze distribution of (*R*)-[^11^C]verapamil into the ventricular system of mouse brain.

Brain uptake was expressed as the brain-to-blood ratio of activity at 25 min after injection of (*R*)-[^11^C]verapamil (*K*_b,brain_). The time point of 25 min was chosen to minimize the contribution of radiolabeled metabolites of (*R*)-[^11^C]verapamil to the PET signal [34]. In scan 1 (before administration of tariquidar), *K*_b,brain_ was low in wild-type (1.3 ± 0.1), *Mrp1^(−/−)^* (1.4 ± 0.1) and *Bcrp1^(−/−)^* mice (1.8 ± 0.1) and high in *Mdr1a/b^(−/−)^* (6.9 ± 0.8) and *Mdr1a/b^(−/−)^Bcrp1^(−/−)^* mice (7.9 ± 0.5) (Fig. 2A,C) suggesting Pgp-selective transport of (*R*)-[^11^C]verapamil at the murine BBB. For substrates of both Pgp and Bcrp1, such as [^11^C]elacridar and [^11^C]tariquidar [21], disproportionally large increases in brain exposure are seen in mice lacking both transporters (*Mdr1a/b^(−/−)^Bcrp1^(−/−)^* mice) relative to single transporter knockout mice (*Bcrp1^(−/−)^* or *Mdr1a/b^(−/−)^* mice), because of a cooperative effect of both transporters in limiting brain entry of dual substrates [35]. For (*R*)-[^11^C]verapamil, *K*_b,brain_ was similar in *Mdr1a/b^(−/−)^* and *Mdr1a/b^(−/−)^Bcrp1^(−/−)^* mice (Fig. 2C) supporting previous evidence that (*R*)-[^11^C]verapamil is not a dual Pgp/Bcrp1 substrate [36]. In response to tariquidar injection during scan 1, a clearly visible rise in brain time–activity curves was observed in mice expressing Pgp (wild-type, *Mrp1^(−/−)^*, *Bcrp1^(−/−)^*) but not in mice lacking Pgp (*Mdr1a/b^(−/−)^* and *Mdr1a/b^(−/−)^Bcrp1^(−/−)^*) (Fig. 2A). This rise in brain time–activity curves was most likely due to inhibition of Pgp at the BBB, resulting in influx of unmetabolized (*R*)-[^11^C]verapamil from blood into brain [6]. Similarly, in scan 2 after tariquidar administration *K*_b,brain_ was significantly (*p* < 0.001) increased relative to scan 1 in Pgp-expressing mice (wild-type: 5.0 ± 0.3-fold, *Mrp1^(−/−)^*: 3.2 ± 0.6-fold and *Bcrp1^(−/−)^*: 4.3 ± 0.1-fold), but not in Pgp knockout mice (*Mdr1a/b^(−/−)^* and *Mdr1a/b^(−/−)^Bcrp1^(−/−)^*) (Fig. 2D), which again supported that (*R*)-[^11^C]verapamil is selectively transported by Pgp. In *Mdr1a/b^(−/−)^Bcrp1^(−/−)^* mice, *K*_b,brain_ was by about 30% decreased relative to scan 1, but this did not reach statistical significance. *K*_b,brain_ values in scan 2 were similar for all mouse types with the exception of *Mrp1^(−/−)^* mice, which had significantly lower *K*_b,brain_ values (*p* < 0.05) than *Bcrp1^(−/−)^* and *Mdr1a/b^(−/−)^* mice (Fig. 2D). The exact reasons for this observation are not known but could be for instance related to lower tariquidar plasma concentrations (which were not measured in the present study) in *Mrp1^(−/−)^* mice. Blood activity concentrations of (*R*)-[^11^C]verapamil were not significantly different among mouse types both in scan 1 and scan 2 and also did not differ in individual mouse types before and after tariquidar administration (Fig. 2E, F).

## 4 Conclusion

Our combined in vitro and in vivo data suggest that verapamil, in nanomolar concentrations as used for PET imaging, is selectively transported by Pgp and not by MRP1 and BCRP at the BBB, which supports the use of (*R*)-[^11^C]verapamil or racemic [^11^C]verapamil as PET tracers of cerebral Pgp function.
